# Fecal transplantation does not transfer either susceptibility or resistance to food borne listeriosis in C57BL/6 and BALB/c/By mice

**DOI:** 10.12688/f1000research.2-177.v1

**Published:** 2013-08-20

**Authors:** Tanya Myers-Morales, Kate M Bussell, Sarah EF D'Orazio

**Affiliations:** 1 Dept. of Microbiology, Immunology & Molecular Genetics, University of Kentucky, Lexington, KY, USA

## Abstract

The composition of the intestinal microbiota has wide reaching effects on the health of an individual, including the development of protective innate immune responses.  In this report, a fecal transplantation approach was used to determine whether resistance to food borne listeriosis was dependent on the murine gut microbiota.  Transplantation of BALB/c/By feces did not increase the susceptibility of C57BL/6 mice to *Listeria monocytogenes* infection.   Likewise, transplantation of C57BL/6 fecal matter did not enhance the resistance of BALB/c/By mice.  Thus, intestinal microbiota composition is not a key factor that confers either susceptibility or resistance to food borne listeriosis in mice.

## Introduction

*Listeria monocytogenes* is a Gram-positive, facultative intracellular bacterial species that can readily adapt to a variety of environmental stresses, including high salt concentration, low pH, and refrigeration ^1^. In humans, ingestion of *L. monocytogenes*-contaminated food results in a wide spectrum of clinical outcomes, ranging from mild, self-limiting gastroenteritis to lethal meningoencephalitis ^2^. The majority of serious infections occur in patients with some degree of immune compromise, but the exact host factors that determine susceptibility to these infections are not well understood.

*L. monocytogenes* infection can be established in almost any outbred or inbred strain of laboratory mice, but there are significant differences among mouse strains for both morbidity and mortality. C57BL/6 mice are one of the most resistant strains, and can readily clear *L. monocytogenes* infection, while BALB/c mice are highly susceptible to developing life-threatening systemic listeriosis. These differences in host susceptibility hold true whether mice are infected by the intravenous route ^3, 4^, via intragastric inoculation ^5^, or by ingestion of contaminated food ^6^.

Genetic analysis of laboratory mouse strains has defined a number of alleles that can be either deleterious or beneficial to mice during *L. monocytogenes* infection ^7– 9^, however, no single trait appears to be entirely responsible for the susceptibility or resistance phenotype. Instead, the ability to clear a bacterial infection is likely to depend upon complex interactions between multiple host factors. It was also recently shown that a novel mechanism, unrelated to specific gene loci, could account for the majority of the difference in host susceptibility to infection between two mouse strains. Willing *et al.* demonstrated that the enhanced susceptibility of C3H/HeJ mice to oral *Citrobacter* infection could be completely transferred to resistant NIH Swiss mice ^10^. To do this, they depleted the gut microbiota of the resistant NIH Swiss mice and repopulated the intestinal tracts of those animals with fecal matter harvested from susceptible C3H/HeJ mice. Their striking results implied that varying gut microbiota compositions could underlie many previously observed differences in host susceptibility to infection with a variety of orally acquired bacterial pathogens.

The mouse strains used by Willing *et al.* came from two different vendors, so it is not surprising that the composition of the gut microbiota in those animals differed significantly. However, the predominant organisms found in the gastrointestinal tract can differ, even for mice housed in the same facility, particularly when the mice are altered in specific components of the immune system. For example, the intestinal microbiota was reported to differ compared with parental control strains in mice expressing human alpha defensin ^11^, or in mice lacking IL-10 ^12^, IL-22 ^13^, neutrophil elastase ^14^, NLRP6 ^15^, or TLR5 ^16^. In fact, it has been shown that the presence or absence of a single type of bacteria in the gut can dramatically alter the development of innate immune responses and inflammatory disease ^17– 19^. In this report, we used the fecal transplantation approach of Willing *et al.* to test the hypothesis that the murine intestinal microbiota can at least partially mediate either the susceptibility of BALB/c/By mice or the resistance of C57BL/6 mice to food borne *L. monocytogenes* infection.

## Materials and methods

### Mice

Female C57BL/6/J (B6) and BALB/c/By/J (By) mice were purchased from The Jackson Laboratory (Bar Harbor, ME) at 4 weeks of age and then housed at the University of Kentucky for 2 weeks in a specific-pathogen free facility with a 14 h light cycle (12 am–2 pm) and a 10 h dark cycle (2 pm–12 am). Groups of four mice were housed in microisolator cages (Inc., Allentown, NJ) lined with coarse grade Sani-Chip bedding (PJ Murphy Forest Products, Montville, NJ). All procedures were approved by the Institutional Animal Care and Use Committee (IACUC) at the University of Kentucky.

### Preparation of fecal matter

Prior to each fecal transfer treatment, fecal pellets were collected from 3–4 donor mice, pooled together and weighed, and then placed in 0.25–1.0 ml of transfer buffer sterile filtered 0.05% cysteine HCl (Calbiochem/EMD Millipore, Billerica, MA) in Dulbecco’s Phosphate Buffered Saline (PBS; Life Technologies, Grand Island, NY), as described previously ^10^. The final volume was adjusted to give 120 mg feces per ml. The fecal pellets were mashed with sterile wooden toothpicks (Wesco Enterprises, Santa Fe Springs, CA), and then vortexed (Vortex Genie; Scientific Industries, Bohemia, NY) at maximum speed for 1 min. The fecal matter was centrifuged (Eppendorf 5417C; Hauppauge, NY) for 3 min. at 800 × ***g*** and the supernatant was used for transplantation into mice.

### Fecal transplantation

**Figure 1.**
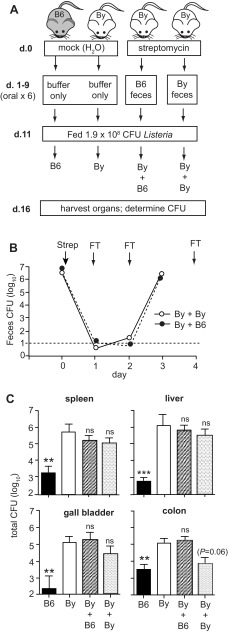
Transplantation of fecal matter from C57BL/6 (B6) mice to BALB/c/By (By) mice does not confer resistance to *L. monocytogenes*. ( **A**) The experimental design for four groups of mice is shown. Mock-treated mice received oral treatments with buffer alone and maintained their original microbiota. Streptomycin-treated mice received a total of 6 fecal transfers over a 9 day period. On day 10, food was restricted and all mice were placed on raised wire flooring for the duration of the experiment. ( **B**) The efficiency of streptomycin pre-treatment was evaluated by monitoring the amount of aerobic CFU shed in the feces. Arrows indicate days of either antibiotic treatment (Strep) or fecal transplants (FT). Mean values ± SEM for groups of 8 mice are shown. ( **C**) Mean values ± SEM (n=8) for total *L. monocytogenes* CFU in spleen, liver, or gall bladder and cell-associated CFU in the colon were determined 5 days post infection. Asterisks indicate mean values significantly different from the mock-treated By group as determined by unpaired t-test. Pooled data from two separate experiments comprising 4 mice per group is shown.

**Figure 2.**
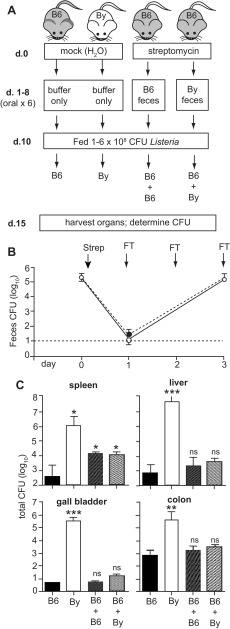
Transplantation of fecal matter from BALB/c/By (By) mice to C57BL/6 (B6) mice does not enhance susceptibility to food borne listeriosis. ( **A**) The experimental design for four groups of mice is shown. Mock-treated mice received oral treatments with buffer alone and maintained their original microbiota. Streptomycin-treated mice received a total of 6 fecal transfers over an 8 day period. On day 9, food was restricted and all mice were placed on raised wire flooring for the duration of the experiment. ( **B**) The efficiency of the streptomycin pre-treatment was evaluated by monitoring the amount of aerobic CFU shed in the feces. Mean values ± SEM for groups of 8 mice are shown. Arrows indicate days of either antibiotic treatment (Strep) or fecal transfers (FT). ( **C**) Mean values ± SEM (n=8) for total *L. monocytogenes* CFU in spleen, liver, or gall bladder and the cell-associated CFU in the colon were determined 5 days post infection. Asterisks indicate mean values significantly different from the mock-treated C57BL/6 group as determined by unpaired t-test. Pooled data (n=8) from two separate experiments (n=4 per group in each) is shown.

The endogenous gut microbiota of 6 week old mice was depleted by treating with a single dose of streptomycin (Sigma-Aldrich, St. Louis, MO). The antibiotic was suspended in sterile water at 500 mg/ml and 50 µl was placed directly in the oral cavity of each mouse. After the oral antibiotic treatment, mice were housed on raised wire flooring (# 3 mesh; Allentown) to prevent coprophagy. Fecal transplants were initiated 24 h later by placing 50 µl of the donor fecal matter directly into the oral cavity of the recipient mice (n=4 per group). Control groups of mice (n=4) were given 50 µl of transfer buffer alone. As shown in Figure 1B and Figure 2B, all mice received fecal transplants 24 and 48 hours after the streptomycin treatment. After the second fecal transplant, the wire flooring units were removed from the cages. Each group of mice (n=4) received a total of four additional treatments over the next 6–7 days (see Figure 1A and Figure 2A), prior to oral challenge with *L. monocytogenes*.

### Quantification of fecal microbiota

Stool samples were collected both before and after streptomycin treatment. Three fecal pellets per mouse were placed in a microcentrifuge tube (VWR; Radnor, PA) containing 1 ml of sterile water. The pellets were mashed with a sterile wooden toothpick, vortexed at maximum speed for 1 min. and centrifuged at 800 × ***g*** for 3 min. The total number of aerobic colony forming units (CFU) was determined by plating serial dilutions of the supernatant on Brain Heart Infusion (BHI) agar (Difco; BD Diagnostics, Franklin Lakes, NJ) and incubating at 37°C overnight.

### Food borne *Listeria* challenge

After completing a total of 6 oral treatments with either donor fecal material or transfer buffer alone, all mice were denied food (but given unrestricted access to water) and placed on raised wire flooring (to prevent coprophagy) for 24 h. Aliquots of intestinally passaged *L. monocytogenes* EGDe InlA ^m^ (generously provided by Wolf Dieter Schubert, Braunschweig, Germany) were prepared and grown as previously described ^6^. Bacteria were washed once with sterile PBS, suspended in 40% melted salted butter (Kroger; Cincinnati, OH) in PBS, and then 5 μl of this mixture was used to saturate a small cube of white bread (Kroger). At the onset of the dark cycle, each mouse was placed in an empty cage with one *L. monocytogenes*-contaminated bread piece and was observed until it picked up the bread and consumed it entirely as previously described ^20^. After ingesting the *L. moncytogenes*-contaminated bread, each mouse was returned to its original cage and was given unrestricted access to mouse chow and water for the duration of the experiment (5 days).

### Quantification of *Listeria* tissue burdens

Five days post-infection, all mice were euthanized by cervical dislocation and the organs were harvested aseptically as previously described ^20^. The total cell-associated *L. monocytogenes* in the colon was determined by extensively flushing the tissue with sterile PBS to remove lumenal bacteria and then homogenizing for 1 min. as previously described ^6, 20^. Spleens and livers were collected in 2 ml of sterile water and homogenized for 30 sec. using a PowerGen 1000 homogenizer (IKA Works, Wilmington, NC). Gall bladders were placed in microcentrifuge tubes containing 1 ml of sterile water, ruptured with sterile scissors (VWR, Radnor, PA), and vortexed at maximum speed for 1 min. Serial dilutions of each tissue homogenate were prepared in sterile water and then plated on BHI/L+G (BHI agar supplemented with 15 g/L LiCl (Sigma-Aldrich) and 10 g/L glycine (Calbiochem), a selective medium that inhibited the growth of intestinal microbiota ^6, 20^. Colony forming units (CFU) were observed after 48 h growth at 37°C and the number of colonies was multiplied by the dilution factor and the total volume of the sample to give the total number of CFU per tissue.

### Statistics

Unpaired t tests were performed using Prism 5 software for Macintosh (Graph Pad). *P* values less than 0.05 were considered significant and are indicated as follows: *, *P* < 0.05; **, *P* < 0.01; ***, *P* < 0.001; ****, *P* < 0.0001; ns, not significant.

## Results

### Fecal transplantation does not transfer the resistance phenotype of C57BL/6 mice to BALB/c/By mice

To test the hypothesis that resistance to listeriosis could be mediated by gut microbiota, fecal matter was recovered from resistant C57BL/6 mice and used to repopulate the intestinal lumens of susceptible BALB/c/By mice ( Figure 1A). Groups of BALB/c mice were given a single oral dose of streptomycin to eradicate the existing microbiota. One day later, the mice began a series of six oral treatments with C57BL/6 fecal matter. The efficacy of the antibiotic treatment was confirmed by plating stool samples from each of the recipient animals. As shown in Figure 1B, the number of bacteria that could be recovered during aerobic growth decreased by more than 5 logs. From 24 to 48 h post-antibiotic treatment, the number of CFU in the stool samples was below the limit of detection (10 CFU) in the majority of the animals ( Figure 1B). By 72 h post-antibiotic treatment, after two fecal transplants, the number of CFU present in the stool samples had recovered to pre-treatment levels. To ensure that the transferred microbiota persisted, each animal received four additional fecal transplants prior to oral *L. monocytogenes* challenge ( Figure 1A).

Groups of BALB/c/By mice transplanted with C57BL/6 microbiota, or repopulated with BALB/c/By fecal matter as a control, were then infected with *L. monocytogenes* via the natural feeding route. The bacterial burdens in the colon, spleen, liver, and gall bladder were determined 5 days post-infection and compared to groups of BALB/c/By and C57BL/6 mice that were mock-treated (no fecal transplantation; see Figure 1A). As expected, C57BL/6 mice had 30 to 1000-fold fewer *L. monocytogenes* in each tissue examined compared with BALB/c/By mice ( Figure 1C). In contrast, there was no significant difference between the groups of BALB/c/By mice that received C57BL/6 fecal transplants and the BALB/c/By mice that were mock treated. Thus, fecal transplantation did not cause BALB/c/By mice to become more resistant to oral *L. monocytogenes* challenge, suggesting that the gut microbiota alone does not contribute significantly to the resistance phenotype observed in C57BL/6 mice.

### BALB/c/By fecal transplants do not render C57BL/6 mice more susceptible to gastrointestinal infection

Susceptibility and resistance to infection are separate traits that are often linked to distinct gene loci. To test the alternate hypothesis that bacteria present in the gut microbiota could confer susceptibility to listeriosis, BALB/c/By feces was transplanted into C57BL/6 mice ( Figure 2A). Groups of C57BL/6 mice were pre-treated with streptomycin and then given 6 oral treatments with BALB/c/By fecal matter. Control groups received C57BL/6 feces or were mock treated ( Figure 2A). Again, the streptomycin treatment efficiently cleared the vast majority of the aerobic bacteria present in the gut lumen within 24 h, and fecal transplantation restored the level of the intestinal microbiota within 3 days ( Figure 2B).

The mice were infected with *L. monocytogenes* by ingestion of contaminated food, and the number of CFU present in the gut and in peripheral tissues was determined 5 days later. Mock-treated C57BL/6 mice had 1000-fold less *L. monocytogenes* than BALB/c/By mice in the colon ( Figure 2C), the site within the intestines which was previously shown to allow for the greatest growth and persistence of *L. monocytogenes* after oral infection ^6^. Transfer of BALB/c/By microbiota did not enhance the ability of *L. monocytogenes* to colonize the colon, as there was no significant difference between the B6 + By group and mock-treated C57BL/6 mice ( Figure 2C).

In the food borne model of listeriosis, the spleen and liver are not colonized until 48 h post-ingestion and bacteria begin to appear in the gall bladder 3–4 days post-infection ^6^. Thus, bacterial burdens 5 days post-infection reflect both the ability of *L. monocytogenes* to disseminate from the gut, and bacterial replication within these tissues. As shown in Figure 2C, resistant C57BL/6 mice had at least 1000-fold fewer *L. monocytogenes* in the spleen and liver and approximately 100,000-fold less bacteria in the gall bladder compared with mock-treated BALB/c/By mice. Transplantation of BALB/c/By fecal matter did not result in an increase in bacterial loads in either the liver or the gall bladder of C57BL/6 mice (B6 + By group; Figure 2C). Fecal transplants did result in a slight increase in the number of *L. monocytogenes* recovered from spleens of C57BL/6 mice, however, a similar increase was observed whether the mice were transplanted with BALB/c/By feces or re-populated with C57BL/6 fecal matter. Together, these results suggest that the gut microbiota of BALB/c/By mice cannot independently enhance susceptibility to food borne listeriosis.

## Discussion

Differing levels of resistance to infection with bacterial pathogens such as *L. monocytogenes* are well documented in mice ^3– 6^. Until recently, it was assumed that specific DNA loci were responsible, and that different combinations of susceptibility and resistance alleles would result in varying degrees of infection in any given mouse strain ^21^. A recent report by Willing *et al.* shattered this paradigm by using a fecal transplantation approach to demonstrate that the gut microbiota alone could confer resistance to *Citrobacter rodentium* infection ^10^. In this study, we used a similar approach to determine whether the composition of the intestinal microbiota could alter the dynamics of *L. monocytogenes* infection in mice. Using a food borne model of listeriosis ^20^, we showed that transplantation of BALB/c/By feces into C57BL/6 mice did not make the mice more susceptible to infection. Likewise, transplantation of C57BL/6 fecal matter was unable to enhance the resistance of BALB/c mice.

A key factor required for *C. rodentium* infection appears to be the induction of a strong Th1 type inflammatory response in the intestines. Lupp *et al.* previously showed that the inflammation itself significantly altered the composition of the gut microbiota in a way that promoted the aerobic outgrowth of both endogenous members of the *Enterobacteriaceae* as well as the introduced pathogen *C. rodentium* ^12^. *L. monocytogenes* is an invasive pathogen, and thus, may not need to overcome colonization resistance or compete with the microbiota for growth in the intestinal lumen in order to establish an infection. *L. monocytogenes* can cross the gut mucosa using M cells or via a “zipper” mechanism of uptake into enterocytes and goblet cells that is dependent on interactions between the bacteria surface protein InlA and E-cadherin on the host cell ^22, 23^. Both mechanisms result in rapid translocation to the lamina propria where the bacteria replicate extensively before disseminating to peripheral tissues to cause systemic listeriosis ^6^. Thus, if even a few *L. monocytogenes* can translocate across the mucosal barrier, the infection will be maintained, even if there is very little growth of the bacteria in the gut lumen.

The fecal transplantation approach is a useful way to significantly alter the gut microbiota, but it does not completely eliminate the native bacterial populations present in any given mouse. A single dose of streptomycin can transiently reduce, by approximately 90%, the density of bacteria recovered using anaerobic culture conditions ^24^, and the intestinal microbiota will return to normal levels within 3–4 days. Willing *et al.* used 16S RNA analysis to show that within 4–5 days of fecal transplantation, mice had an altered microbiota composed of microbial families from both the donor and recipient mouse strains, with ratios that strongly reflected the donor strain ^10^. Given these parameters, the fecal transplantation approach is most informative if the presence of a transferred microbial species confers a particular phenotype, even when the original microbiota is not completely displaced. Thus, we cannot rule out the possibility that small numbers of native microbial species not eliminated by streptomycin treatment could confer some degree of susceptibility to BALB/c/By mice or resistance to C57BL/6 mice. However, the results shown here clearly suggest that the composition of the gut microbiota is not a major factor that governs susceptibility to food borne listeriosis, as is the case for *C. rodentium* infection in mice.
