# Supplementary material for: Fecal transplantation does not transfer either susceptibility or resistance to food borne listeriosis in C57BL/6 and BALB/c/By mice
Source: F1000Res. 2013 Aug 20;2:177. [Version 1] doi: 10.12688/f1000research.2-177.v1 (PMC3869484; doi:10.12688/f1000research.2-177.v1)
Supplement: Peer review history

# Peer review: Referee response for version 1

**Published:** 2013-11-19

**Authors:** Laurel Lenz

**Affiliations:** 1 Integrated Department of Immunology, University of Colorado, Denver, CO, USA

Myers-Morales *et al.* present results indicating that B6 or BALB mice depleted of gut flora using streptomycin do not show altered resistance when given a fecal transplant from the opposing strain. The studies suggest, but do not prove, that gut flora do not contribute to the differing resistance of these strains.

A few concerns, if addressed, would strengthen the paper.

1. It is assumed that B6 and BALB mouse strains have differences in their streptomycin-resistant flora. However, this was not actually established by the authors.
   
2. A control group with streptomycin treatment alone might establish whether depletion (not "eradication," as stated) of the flora itself affected resistance. If so, the reconstitution apparently restored the original phenotype regardless of donor. This or other more direct approaches could be used to establish whether the transfer was successful at modifying the flora in recipients.

I have read this submission. I believe that I have an appropriate level of expertise to confirm that it is of an acceptable scientific standard.

# Peer review: Referee response for version 1

**Published:** 2013-08-27

**Authors:** Benjamin Willing

**Affiliations:** 1 Agricultural, Food, and Nutritional Science, University of Alberta, Edmonton, T6G 2P5, Canada

The article “Fecal transplantation does not transfer either susceptibility or resistance to food borne listeriosis in C57BL/6 and BALB/c/By mice” by Myers-Morales *et al*. demonstrates that the intestinal microbiota is not a major determinant in the differences in susceptibility between C57BL/6 and BALB/c/By mice. It is an important report of negative results.   

 A couple of clarifications are requested/suggested.  

 1)    While not necessary, the characterization of the microbial community in transplanted mice to confirm effective transfer would strengthen the claim. 

 2)    The report by Willing *et al*. demonstrated that susceptibility in C3H/HeJ mice could be partially transferred to resistant NIH Swiss mice, not completely. While higher colonization and increased pathology was shown, the transfer of mortality phenotype was not demonstrated.

 3)    The presentation of data for CFU counts should be normalized to weight. (CFU/g). CFU/organ or CFU/fecal pellet would also be acceptable. 

 4)    In the results section: Streptomycin depletes, but does not “eradicate” the microbiota.

I have read this submission. I believe that I have an appropriate level of expertise to confirm that it is of an acceptable scientific standard.
